# The EAES intellectual property protection guide

**DOI:** 10.1007/s00464-025-12173-7

**Published:** 2025-10-22

**Authors:** Kiyokazu Nakajima, Luigi Manfredi, Etsuko Yoshida, Tim Horeman, Yoav Mintz

**Affiliations:** 1 Department of Next Generation Endoscopic Intervention (Project ENGINE), Graduate School of Medicine, The University of Osaka, Suite 0802, BioSystems Bldg., 1-3, Yamadaoka, Suita, Osaka 565-0871 Japan; 2 Respiratory Medicine and Gastroenterology, School of Medicine, University of Dundee, Dundee, UK; 3 Faculty of Intellectual Property, Osaka Institute of Technology, Osaka, Japan; 4 Department of Biomechanical Engineering, Delft University of Technology, Delft, The Netherlands; 5 Department of General Surgery, Hadassah Hebrew University Medical Center, Jerusalem, Israel; 6 Faculty of Medicine, Hebrew University of Jerusalem, Jerusalem, Israel

**Keywords:** Intellectual property, Patent, PCT, Invention by employees, Nondisclosure agreement, EPO

## Abstract

This guide is designed to assist surgeons in understanding the proper procedures for legitimately securing the outcomes of their original ideas and collaborations with industry partners as “intellectual property.” Our web-based survey conducted among EAES members in 2019 revealed that surgeons have traditionally shown limited interest in intellectual property. The findings suggested that even when surgeons generate and realize novel concepts, these innovations are often inadequately protected, leaving them vulnerable to misappropriation. Accordingly, this guide focuses on the patenting process for surgeons engaged in collaborative research with industry. It addresses common questions that arise during this process in a Q&A format. The guide also explains key aspects of communication with corporate partners and intellectual property specialists during patent application procedures. Furthermore, it outlines the transition from domestic to international patent protection, with particular emphasis on the Patent Cooperation Treaty (PCT) system. Although this article serves as a practical guide for surgeons seeking to protect their intellectual contributions, it may also be used as an educational resource for industry designers, engineers and business developers, highlighting the importance of respecting and safeguarding the intellectual property rights of medical professionals.

## Introduction

### Backgrounds

In the recent years, collaboration between surgeons and industry has become a cornerstone of surgical innovation. In particular, efforts to develop medical devices through such collaborations are widely recognized as crucial for promoting medical advancements, enhancing safety, and improving patient outcomes.

To foster industry–academia collaboration, it is essential to have a proper understanding of intellectual property (IP). The essence of IP is not to hinder collaboration between universities, medical professionals, engineers, and industry but rather to facilitate and protect it. Unfortunately, due to a lack of knowledge about IP, many medical professionals and engineers involved in the research and development (R&D) of medical devices have forfeited valuable IP rights without realizing the missed opportunities. Mishandling IP rights raises concerns that promising medical device R&D may not lead to commercialization.

To ensure that valuable research efforts result in successful product releases, these innovations need to be properly protected by patents. The R&D team—including medical professionals, engineers, and support staff—should possess a fundamental understanding of IP. This document aims to guide EAES members by providing essential knowledge on IP to increase the likelihood of successfully bringing innovative medical devices to market while safeguarding their rights.

### Current status of IP rights protection in the Medtech field

Surgeons are inherently creative. In the operating room, they use various surgical tools much like craftsmen, yet they are often not entirely satisfied with the design or performance of these instruments. Many surgeons, whether early-career or highly experienced, have excellent ideas for solving existing device issues and improving their functionality. However, most do not know how to turn their ideas into reality.

Our engineering partners are also highly creative. While they may lack medical expertise, they understand the physics behind device function and, with proper guidance, can design working prototypes. Many engineers are enthusiastic early adopters of innovation. As long as the concept is communicated effectively, they can create remarkable prototypes.

The critical questions that remain are: Are these valuable ideas from both surgeons and engineers being properly developed? Or are they being lost without utilization? Are these ideas being transformed into products by someone else without your knowledge? Are your ideas adequately protected?

A web-based survey conducted by the EAES Technology Committee in 2019 revealed that over 70% of EAES members were "dissatisfied" with current medical devices [1]. In addition, as expected, 66% of respondents had specific ideas for new or improved devices to address unmet needs. However, surprisingly, 60% did not take any steps to protect their ideas before sharing them with industry, publishing in academic journals, or presenting at conferences [1]. These actions often resulted from the lack of education on IP rights in medical training programs.

Based on the EAES survey results, we recognized the urgent need to take measures to protect our members’ IP rights, which may be unintentionally lost through their daily research and clinical practice. Although surgeons and engineers do not need to become IP specialists, they should have a basic understanding of IP rights. This guide provides foundational knowledge to help protect your ideas and prevent them from being misused or overlooked.

## Terminology

### Patent

An invention refers to a novel idea or method that did not previously exist.

A patent is one of the most important IP rights, as it grants the inventor the exclusive right to use the invention for a specified period. This means that others cannot use the invention for commercial purposes without permission [2, 3].

No matter how brilliant an idea (invention) may be, if the inventor keeps it to themselves, it may never materialize or achieve widespread adoption. The patent system protects inventions by granting exclusive rights for a certain period, provided they meet criteria such as novelty and an inventive step. This system also promotes further technological development by preventing redundant research and development making patented inventions publicly available.

A patent is granted if:

1. It describes a novel idea or method that has not been patented before.
2. It is non-obvious.
3. It does not involve a law of nature.
4. It has not been previously disclosed in the public domain.
5. If it is technically applicable

A patent will not be granted if:

1. It describes a law of nature or a natural phenomenon, as these belong to humanity. For example, Newton’s law of gravity or Einstein’s energy equation (E = mc^2^) cannot be patented.
2. It is an artistic creation, such as a painting or sculpture.
3. It involves a skill-based method, such as a golf swing technique.
4. It includes a new substance existing in nature, which is merely a discovery.
5. Its technical working principle is not clear. For example, a teleportation machine cannot be patented.

In addition, for an invention to be patented, it must meet criteria such as novelty and an inventive step, ensuring that long-known public knowledge is not later monopolized by a single individual. Medical devices can be patented if they meet the above conditions, but it is important to note that each global jurisdiction has its specific conditions for patenting medical methods such as surgical procedures and diagnostic methods (Table 1).

**Table 1:** Patentability of medical methods and devices (US / EPC / JP / CN)

Jurisdiction	Therapeutic methods (e.g. surgical procedure)	Diagnostic methods	Medical devices	Notes
United States	Excluded from patentability if directed to physicians (35 USC §287(c))	Patentable if not solely mental steps; restrictions apply	Patentable	Enforcement against medical practitioners limited
European Union (EPO)	Explicitly excluded under EPC Art. 53(c)	Diagnostic methods performed on the human/animal body excluded; in vitro methods patentable	Patentable	Clear exclusion for medical methods
Japan	Excluded from patentability (Patent Act Art. 29(1), interpreted with Art. 32)	Excluded if practiced on the human body; in vitro diagnostic methods patentable	Patentable	Medical methods are treated as unpatentable inventions
China	Medical methods (diagnosis and treatment) are not patentable (Patent Law Art. 25)	Excluded if performed on the human/animal body; in vitro methods patentable	Patentable	Devices and pharmaceuticals patentable, but methods excluded

### Design

The aesthetic appearance of an industrial product- including its shape, pattern, or color—can be registered as a “design right.” As with patents, a design must meet specific criteria, such as novelty, to qualify for registration. Industrial designs must be suitable for mass production and cannot include unique works of art [2, 3].

### Trademark

A trademark is a unique symbol or word(s) representing a product. Once issued, a trademark prevents others from using the same mark. If a product name is followed by “TM” (Trade Mark), it signifies an unregistered claim to the mark. Once formally registered with the appropriate authority, the symbol “®” (registered) can be used. In Europe, pending trademarks may be followed by “EUTM” (European Union Trade Mark), while officially registered marks, by the European Union Intellectual Property Office, are labeled “RCD” (Registered Community Design). Unlike patents, trademarks do not expire as long as they are actively used.

### Copyright

"Copyright" is the right granted to the creator of a work (the author) to prevent others from copying or using the work—such as on the Internet—without permission. If someone else wants to use the work, the author can grant permission under certain conditions or refuse the use, except in specific cases where the right is limited. A work refers to the creative expression of an idea or emotion; however, copyright typically does not protect the idea itself, procedures, methods of operation, or mathematical concepts. Copyright related to visual material can be managed by using a Creative Commons license as is an alternative way to alert users to what they can or cannot do with your images of your idea and provide means to put your label on your work.

### European Patent Office (EPO)

**Fig. 1:**
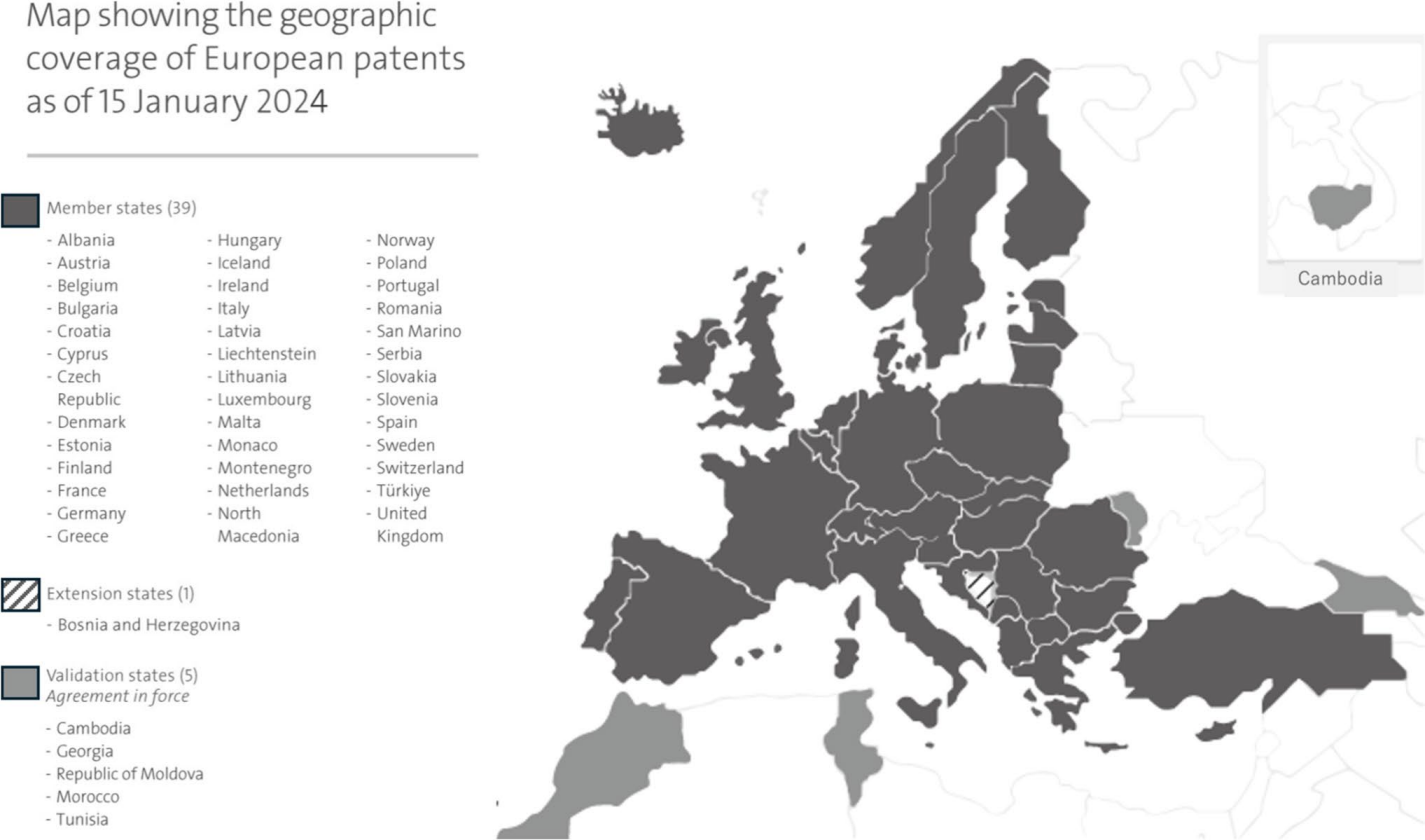
Map showing the current geographic coverage of European patents. *Source*: https://www.epo.org/en/about-us/at-a-glance#&gid=1&pid=1

The European Patent Office (EPO), established in 1973, examines European patent applications, enabling inventors, researchers, and companies to secure patent protection across up to 45 countries with a single application (Fig. 1) [4]. These countries include the 27 European member states, one extension state, and 5 validations states. The EPO accepts applications in English, French, or German.

### European Union Intellectual Property Office (EUIPO)

The European Union Intellectual Property Office (EUIPO), based in Alicante, Spain, oversees the registration of EUTM and RCD (see 2.3.). Formerly known as the Office for Harmonization in the Internal Market (OHIM), it was renamed in 2016.

### Patent Cooperation Treaty (PCT) application

A PCT application allows for a single international patent application to serve as the basis for seeking patents in multiple member states [2, 3]. As of 2024, the PCT includes 157 countries. This system streamlines the filing process and secures an official filing date across participating countries, saving time and reducing complexity [5]. However, within 30 months of filing, applicants must enter the "national phase" in each country where they seek patent protection.

### Inventions by employees

Universities, research institutes, and companies often have regulations governing employee inventions. Typically, inventions conceived during employment belong to the employer due to the organization’s investment in research facilities, personnel, and salaries [2, 3].

However, depending on the country, if an invention is unrelated to an employee’s work (e.g., a surgeon devising an innovative method for stocking supermarket shelves), the IP rights may belong solely to the inventor. If an employer declines to participate in the patenting process, their claim to ownership can be reduced or relinquished.

### Nondisclosure agreement (NDA)

**Fig. 2:**
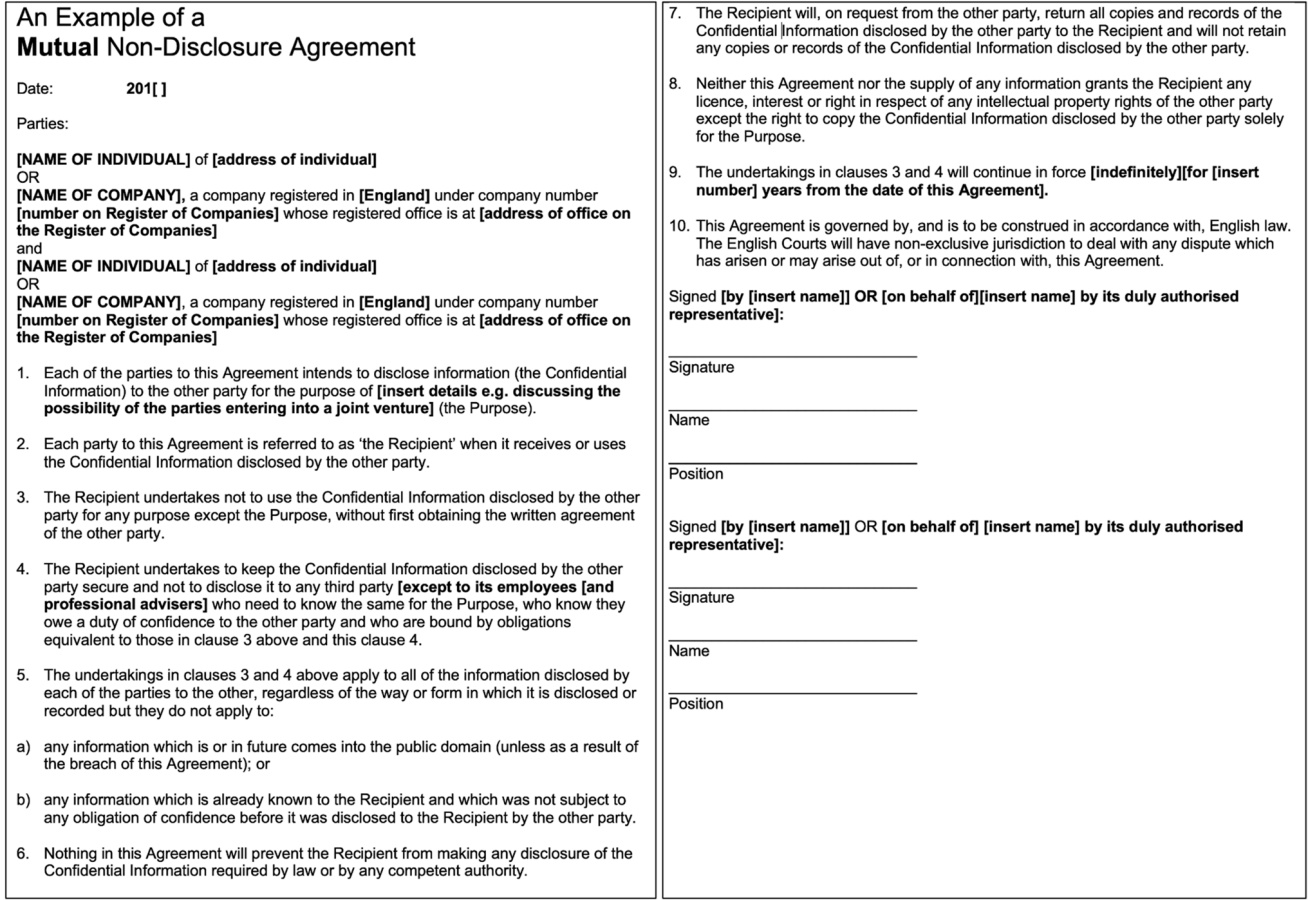
Example of mutual-nondisclosure agreement

Before disclosing an invention to a third party, an NDA (also called a Confidential Disclosure Agreement, CDA) should be signed (Fig. 2) [6]. Without an NDA, ideas may become public knowledge, making patent protection impossible. If you and your employer decide to engage in a joint R&D with a company without signing an NDA, a “joint research agreement” should be in place, and the rights of both parties should be respected. Keep in mind that in this case, the company involved is just a third party to you, therefore, sharing ideas is risky.

Can you disclose your ideas to a personal friend who is part of a company dealing with the same field of invention? Although you have a close friendship or even a familial relationship, without an NDA, disclosure of your ideas could be considered public knowledge once you do. The novelty of the invention may be lost which will be a major obstacle for obtaining a patent [1, 2].

Researchers have a natural desire to share their ideas with others [1]. In fact, situations in which you “want to showcase your idea” to a company do not necessarily occur only when you actively approach a company. It can also occur, when you attend a “new product presentation” by a company. If you share your ideas on how to improve the product presented, you may lose the value of your idea. Unfortunately, most briefings do not include a contract that protects your IP. This is why if you think you have an idea that would significantly improve the product, you should first present this to your Tech Transfer office and protect your idea by signing and NDA before presenting it to others [6].

If the company is not willing to sign an NDA it is best to avoid a joint R&D with such a company.

## IP Rights Protection

### Basics

#### What are the benefits of obtaining a patent?

Once you obtain a patent, you will have the exclusive right to use the patented invention for financial gain over a set period. This means you can prevent others from using your invention for profit without your consent [2].

A patent also allows you (or the university or research institute to which you belong) to license the invention to a third party for commercial use [6]. In such cases, a "license agreement" is provided, allowing you to receive a portion of the revenue as a "license fee." Typically, royalties are calculated as a percentage of net sales and are specified in the contract.

Beyond these financial benefits, patents serve as a valuable asset for researchers. Like academic publications, patents are published in patent gazettes and widely disseminated within the industry. In recent years, IP has become an important metric of a researcher's achievements, often equated with or even surpassing academic publications in significance. IP, along with research capabilities and infrastructure, forms the foundation of "research seeds," and researchers with a strong IP portfolio are more competitive in securing funding.

### How much income can I earn from a patent?

For researchers, income from patents comes in the form of "royalty payments" when patents are licensed to companies. However, patents themselves do not generate revenue unless they are sold or their rights are transferred for a fee. Selling a patent outright may not be the best choice, as it often yields only a fraction of the potential profit the patent could generate over time. The decision to sell a patent should be weighed against the risk of failing to bring a product to market successfully.

Revenue from patents is generally calculated as net sales multiplied by the royalty rate. Therefore, the income depends on the market success of the product incorporating the patented invention. In highly profitable industries with few competing technologies, a new product based on your patent could become a major commercial success.

As mentioned earlier, patent rights are typically owned by the university or institution, not the individual researcher [2]. Royalty income is then divided between you and your institution based on your employment contract. While many institutions have fixed royalty distribution policies, if your invention is groundbreaking, you may attempt to negotiate better terms with your employer. Your institution's technology transfer office (TTO) plays a crucial role in patenting and commercialization, but it relies on your research and expertise. Without your continued involvement, the chances of successfully transforming a patent into a marketable product are slim.

### How much time, effort, and expense does it take to obtain a patent?

A patent application remains confidential for 18 months after filing [2, 3]. During this period, applicants should conduct searches for similar patents and engage with potential commercial partners.

**Fig. 3:**
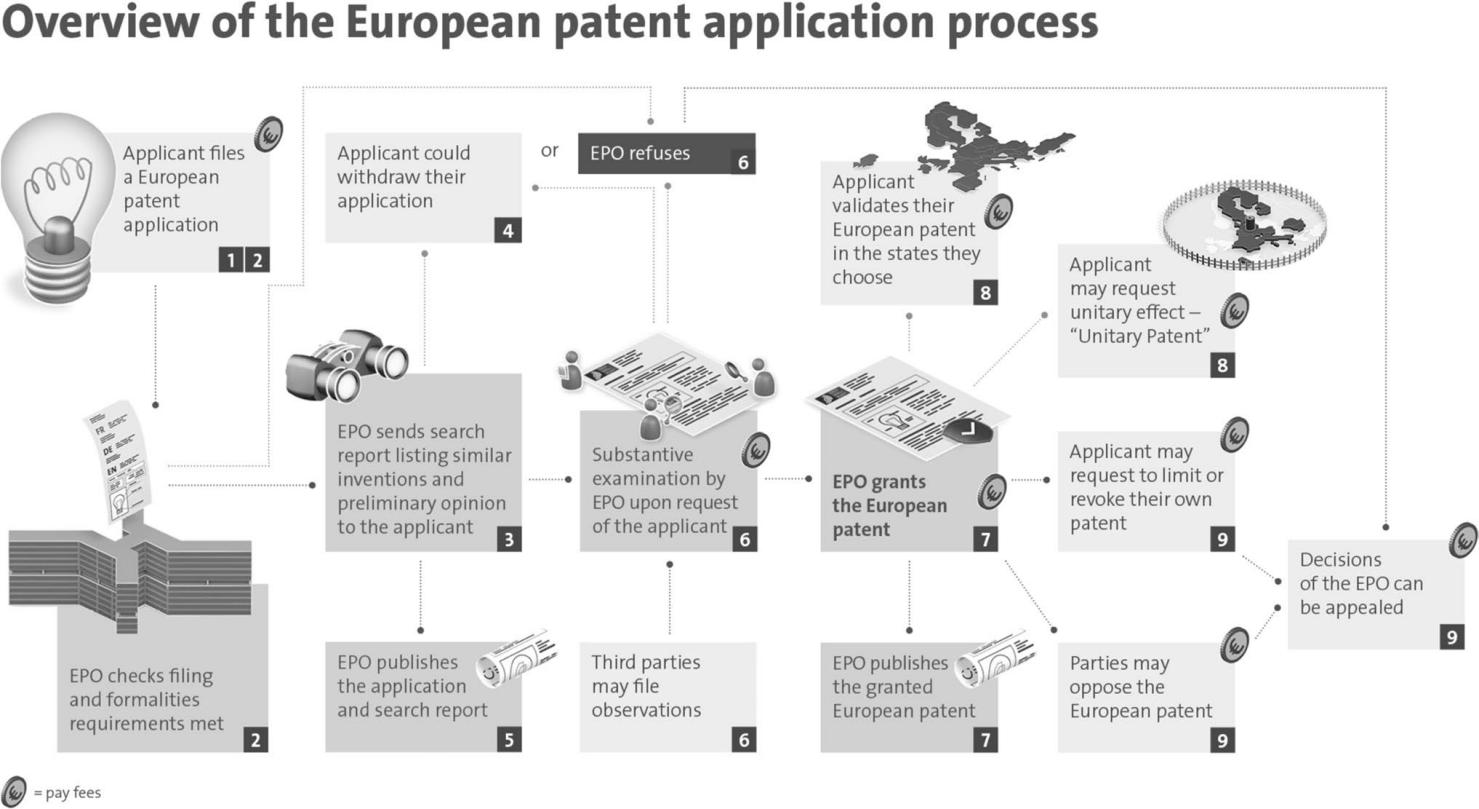
Overview of the European patent application process. *Source*: https://www.epo.org/en/new-to-patents/how-to-apply-for-a-patent#&gid=1&pid=1

The next step is to request an examination, which varies by country [3]. If no request is made, the application is considered “withdrawn.” Once requested, a government examiner conducts a substantive examination to determine patentability (Fig. 3). Examiners often issue objections, requiring applicants to respond with clarifications or modifications. This back-and-forth can prolong the process and increase costs. If the examiner ultimately approves the application, a patent is granted upon payment of the registration fee. Otherwise, a rejection is issued. The first examination result typically arrives 1.5 to 3 years after filing.

In terms of costs, a PCT application requires approximately €1,840—€3,800 for filing, examination, and registration fees [5]. Additionally, hiring a patent attorney adds to the expense, as managing a patent application independently is impractical due to its complexity.

### How long is a patent valid? What happens when it expires?

Patent rights generally last for 20 years from the filing date [2]. However, patents can expire earlier if the annual maintenance fees (“annuities”) are not paid. Other circumstances, such as abandonment or the lack of legal heirs, can also lead to patent expiration.

Once a patent expires, the invention enters the public domain, meaning anyone can use it freely [2]. A common example is generic drugs. Once the patent on an original drug expires, other companies can manufacture and sell identical medications without restriction.

### Patent ownership

#### Who owns the patent?

Patent rights do not necessarily belong to the “inventor” but to the “applicant” who files for the patent [3]. The inventor is the person who conceived the invention, whereas the applicant is the entity that holds the right to obtain the patent. Typically, universities or companies apply for patents rather than individual researchers.

In cases of employee inventions, institutions automatically acquire the “right to obtain a patent” for discoveries made during the course of employment. As a result, the researcher cannot file an application independently, and the institution becomes the patent owner.

Under prevailing legal frameworks, artificial intelligence (AI) is not recognized as a legitimate inventor in the majority of jurisdictions. Nevertheless, the rapid advancement of AI technologies is increasingly challenging traditional notions of inventorship and prompting a reevaluation of existing IP laws and regulations [7].

### Do I own a new surgical tool idea I conceived during surgery?

While the initial idea is yours, under the “employee invention” principle, innovations developed during your work generally belong to your employer. Think of the operating room as a research facility and the surgical team as collaborators—without these resources, you may not have arrived at your idea. The same logic applies to laboratory research, where prior findings from colleagues contribute to discoveries.

Since the employer facilitates the invention process, it is typically entitled to a share of any benefits. If the institution claims ownership of the invention, the employee is entitled to reasonable compensation. This way, the interests of both the employer and the inventor are harmonized.

### If I come up with an idea outside working hours, does it belong to me?

Not necessarily. The key factor is whether the idea relates to your job. If, as a laparoscopic surgeon, you conceive a “new surgical clip” at home, it would still be considered an employee invention and belong to your institution. However, if you develop a “new cooking utensil,” unrelated to your work, it remains your personal intellectual property. Obviously, the question whether and how your idea is linked to your job can lead to complicated discussion so be aware of this.

### Disclosing ideas

#### Should I keep my ideas to myself?

No. Keeping inventions secret increases the risk that valuable technologies will remain unused. For the greater good, you should actively explore commercialization avenues.

If your institution has an IP department or TTO, consult them first. They will help mature your idea into a patent and facilitate licensing agreements. To initiate this process, you will be asked to file a Disclosure of Invention (DOI), detailing ownership, potential applications, and commercial prospects. Resolving ownership issues early in the process helps prevent disputes later.

### Can I talk to my colleague?

Yes, if they are within your institution, as internal confidentiality obligations typically apply. However, discussing your idea with external collaborators without a signed NDA risks compromising your invention’s novelty.

Joint research agreements often include confidentiality clauses, but in medical collaborations, the focus is often on publication rights rather than IP protection. While professional courtesy exists among doctors, formal agreements are essential to avoid conflicts over IP ownership. Generally, it is advised to consult your IP department or TTO before talking to colleagues either in your institution or outside of it. If a colleague will support your invention or add to it, you may need to adjust the DOI or file a new one.

### Should I report to my supervisor?

Yes. Reporting ensures proper IP protection and prevents inadvertent disclosures. A well-informed team can collaboratively nurture and safeguard IP. Since commercializing a patent requires extensive teamwork, securing internal support is crucial.

### Can I present my idea at a medical conference?

No, not before filing a patent application. Even a theoretical presentation or abstract counts as public disclosure, making it impossible to secure a patent later.

### If I publish my idea in an academic journal, will it be protected by copyright?

No. Copyright protects “works” (e.g., articles, books) but not technical ideas. Once an idea is published, it enters the public domain and loses patent eligibility [2]. File a patent application before submitting a paper.

### Can I obtain a European patent if I have already published my work in a U.S. journal?

No. Most developed countries follow a global standard: prior publication anywhere in the world constitutes “prior art,” voiding the novelty requirement for patents. Conference presentations and journal publications, whether domestic or international, should always be approached with IP protection in mind [2, 6].

Table 2 shows a checklist on how to align academic goals (presenting at congress, publishing on journal) with IP strategies. Table 3 depicts recommendations for institutional policy on disclosing research-generated ideas.

**Table 2:** Checklist alignment academic goals with IP strategies

1. Identify potential IP early	
Review research for novel, useful, and nonobvious findings	
Consider potential commercial, industrial, or societal applications	
Understand and clarify why potential IP licensees should be interested	
2. Document and record contributions	
Keep lab notebooks and detailed records for patent support	
Clarify inventorship (not always the same as authorship)	
3.Investigate on IP ownership	
Check if the IP is created within a consortium or as result of a grant or external funding	
Check and understand how potential IP is claimed by potential partners within contracts	
4. Submit an Invention Disclosure Form (IDF)	
Notify your institution’s Technology Transfer Office (TTO) or IP office	
Include details about the invention, contributors, and funding sources	
5. Delay Public Disclosure Until Idea is filed and a priority date is established	
Do **not** publish, present, or post preprints before TTO review	
Understand that public disclosure before filing a patent may forfeit rights	
6. Coordinate patent filing and academic deadlines	
Align patent applications with conference or journal submission dates	
Work with the TTO to ensure filing is done **before** public release	
7. Maintain Communication with the TTO	
Inform the office about planned presentations, posters, or thesis submissions	
Get clearance if unsure about whether the work includes protected IP	
Be ready to provide drawings or additional information when needed	
8. Educate research team members	
Train students and collaborators on the importance of IP timing	
Discuss how publication and patenting can complement or hurt each other

**Table 3:** Institutional policy recommendations: managing research-generated ideas

Early identification and disclosure	
Mandate invention disclosure prior to any public dissemination (publications, presentations, thesis submissions)	
Require researchers to submit invention disclosure forms as part of research project wrap-up or grant reporting	
Encourage IP awareness in research proposal design, especially for funded projects	
Pre-publication IP review process	
Implement a mandatory IP screening of research outputs (papers, posters, abstracts) before submission	
Establish a fast-track review process to avoid delays in publication	
Provide clear guidelines on what constitutes public disclosure and its risks for patentability	
Clear ownership and inventorship guidelines	
Define ownership of IP developed using institutional resources (usually belongs to the institution or is defined in the consortium or funding contract)	
Clarify inventorship versus authorship and ensure proper legal attribution	
Include IP clauses in employment contracts and research collaboration agreements	
Researcher support and training	
Offer regular IP training workshops for faculty, staff, and students	
Create concise guides or flowcharts on how to handle IP in research	
Provide advisory support through the Technology Transfer Office (TTO) or equivalent	
Commercialization and incentives	
Define processes for patenting and licensing, led by the TTO	
Ensure revenue-sharing mechanisms are transparent and fair (e.g., percentages to inventors, departments, and central administration)	
Support startups or spin-offs through incubators or legal/financial advising	
Collaboration and funding agreements	
Include IP terms in sponsored research agreements, especially with industry partners	
Use standard templates for Material Transfer Agreements (MTAs), Nondisclosure Agreements (NDAs), and Collaborative Research Agreements	
Address joint ownership and dispute resolution mechanisms in multi-institutional collaborations	
Governance and oversight	
Establish a Research IP Committee to advise on complex IP issues or disputes	
Monitor compliance with IP policies and track performance metrics (e.g., disclosures filed, patents granted)	
Review and update policies regularly to adapt to changing legal and research landscapes	

### Collaborating with industries

#### Can I contact an engineer I know personally to create prototypes of my idea?

No. Never share your idea with anyone who has not signed an NDA regarding the development of the device, unless you consider them a joint inventor with appropriate rights to your invention (see 2.9). The (financial) consequences of breaking the NDA should be well described and based on a concrete legal foundation.

Check if there is an NDA between you (or your institution) and the company regarding the development of that device. If no such agreement is in place, your act of communication is considered “public knowledge” to a third party, and you will lose the novelty of your invention. Even if you have shared the information with only one person, it is still considered public knowledge if that person is not obligated to maintain confidentiality. This rule applies regardless of your personal relationship with them.

Furthermore, it is advisable not to disclose your invention via email, even to a party bound by confidentiality obligations. This mode of communication risks exposure to third parties who may not be bound by confidentiality agreements, potentially leading to public disclosure. When communicating with collaborators or patent firms, ensure that all shared documents are password-protected.

### How can I start a joint R&D project with a company?

**Fig. 4:**
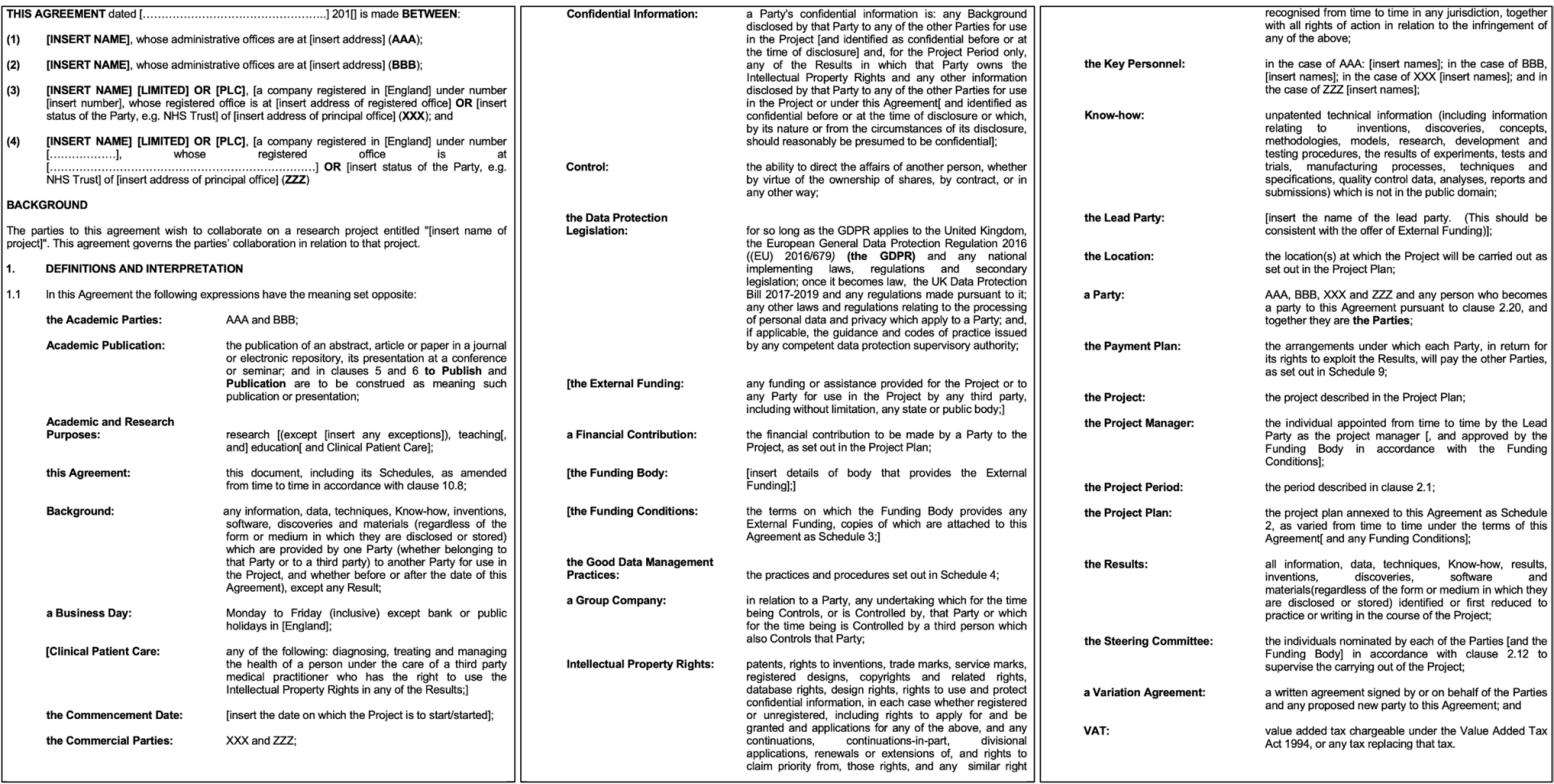
Example of joint research agreement form (partial). For the full document, https://www.gov.uk/government/publications/non-disclosure-agreements [8]

It is advisable to establish a “Joint Research Agreement” to define in advance the sharing of R&D expenses and the handling of any results obtained (Fig. 4) [8].

If a company expresses interest in the new R&D theme you propose and decides to conduct joint R&D, you should go a step beyond the NDA (see 2.9) and formalize a “Joint Research Agreement.” This agreement should not be between you personally (or your research team) and a specific individual at the company (or their department) but between your organization (e.g., university, hospital) and the company. If your organization has an IP department, they will handle much of the paperwork for you. Many institutions have pre-drafted agreement templates (see Appendix). If the company also has an IP department, negotiations will typically be conducted between the IP departments of both parties.

Key elements of a joint research agreement include:

1. Cost sharing
  The agreement should clearly outline how both parties will share the costs associated with R&D. Typically, the company covers all or part of the research costs, while the researcher contributes intellectual input, ideas, research facilities (such as laboratories), and human resources.
2. Handling of results
  The agreement should specify how research results will be managed before any actual results are obtained. If the results are novel and subject to patent application, questions will arise regarding who will file the application, who will bear the costs, and who will be responsible for maintaining the patent. Since it is impossible to predefine the inventor or the specific content of the application, it is common to include a clause stating that “both parties will consult with each other before filing the application.”

### Patent application process

#### I have an idea for a device that seems very new, but has it already been patented?

Conduct a “prior art search.” For an idea to be patentable, it must:

1. Be novel, meaning it is not identical to a previously known invention (prior art).
2. Possess an inventive step, meaning it is not an obvious improvement that anyone skilled in the field could easily derive from prior art.

Most researchers become aware of past inventions only after they have developed something new. Investigating existing inventions is called a prior art search, which includes searching patent databases and academic literature. Free online tools such as Google Patents and the EPO database can help:

- http://ep.espacenet.com/ – Contains patents from over 90 countries, including European, U.S., and Japanese patents.
- PATENTSCOPE, by WIPO (World Intellectual Property Organization), – Provides access to PCT international applications and patent information from over 80 countries. https://patentscope2.wipo.int/search/en/search.jsf

For a more comprehensive search, hiring a patent firm is recommended. These firms conduct detailed searches tailored to specific cases, providing reliable results. The cost will vary depending on the complexity of the search. Your role is to review the identified patents and determine whether your idea offers sufficient novelty.

### I don't have a prototype yet. Can I get a patent based only on an idea and theory?

Yes. If your invention can be clearly explained with drawings, you can obtain a patent even without a prototype.

For mechanical or medical devices with unique structures or combinations of components, a patent can be granted based solely on an idea, provided that the invention is sufficiently detailed at the time of application. If your drawings clearly depict how the device can be manufactured, operated, and function, the invention is considered “complete.” However, if the feasibility of the device is uncertain without building and testing a prototype, it is considered incomplete.

Nonetheless, early prototypes are highly recommended. Even if they do not function perfectly, they help illustrate your idea. If you create a working prototype, take photographs and videos immediately and document test data. This evidence strengthens your patent application and serves as a valuable tool when approaching companies for R&D collaborations.

### How specific must an idea be to qualify for a patent?

The idea must be sufficiently concrete and complete. In medical devices, providing detailed drawings explaining structure and function is usually sufficient. In pharmaceuticals, biotechnology, and chemistry, experimental results are crucial. For instance, patent applications may be rejected if efficacy is demonstrated only in vitro but not in vivo.

In any case, “concrete” means the invention must be developed to a level where it can be industrially applied [2]. For medical devices, this means detailed schematics; for pharmaceuticals, it requires validated experimental data. An invention lacking these elements is deemed incomplete and ineligible for patent protection.

### My research is advancing. When should I apply for a patent?

Consider applying for a patent when your idea is sufficiently concrete and complete or about to be completed. Although it is possible to apply for a patent when you only have an idea, most cases are preceded by joint R&D, evaluation of prototypes, and accumulation of experimental data. No matter how brilliant an idea may be, an unfinished invention that lacks specificity will not be considered eligible for protection.

### My institution does not have IP department. How can I get help?

If your institution, does not have an IP department, consult with a patent attorney. A “patent attorney” is a person who holds a national license to practice patent, trademark, and other industrial property rights. Your confidentiality is assured because the patent attorneys are obligated to maintain confidentiality.

### International patents

#### What is an international patent? What are its advantages?

In these days of rapid globalization, securing patent protection in your country is not sufficient. To enable sales and overseas markets, it is imperative to secure patent protection in countries targeted for marketing and sales. An international patent application under the PCT simplifies the process of seeking patent protection in multiple countries [2, 3, 6]. The PCT allows a single application to be recognized across all PCT member states, establishing an official filing date in each country. This saves time, money, and the complicated procedures involved in filing an international application.

However, the PCT application only standardizes procedural aspects; it does not grant a universally valid patent. To secure patents in individual countries, you must enter the national phase of each country within 30 months of filing.

**Fig. 5:**
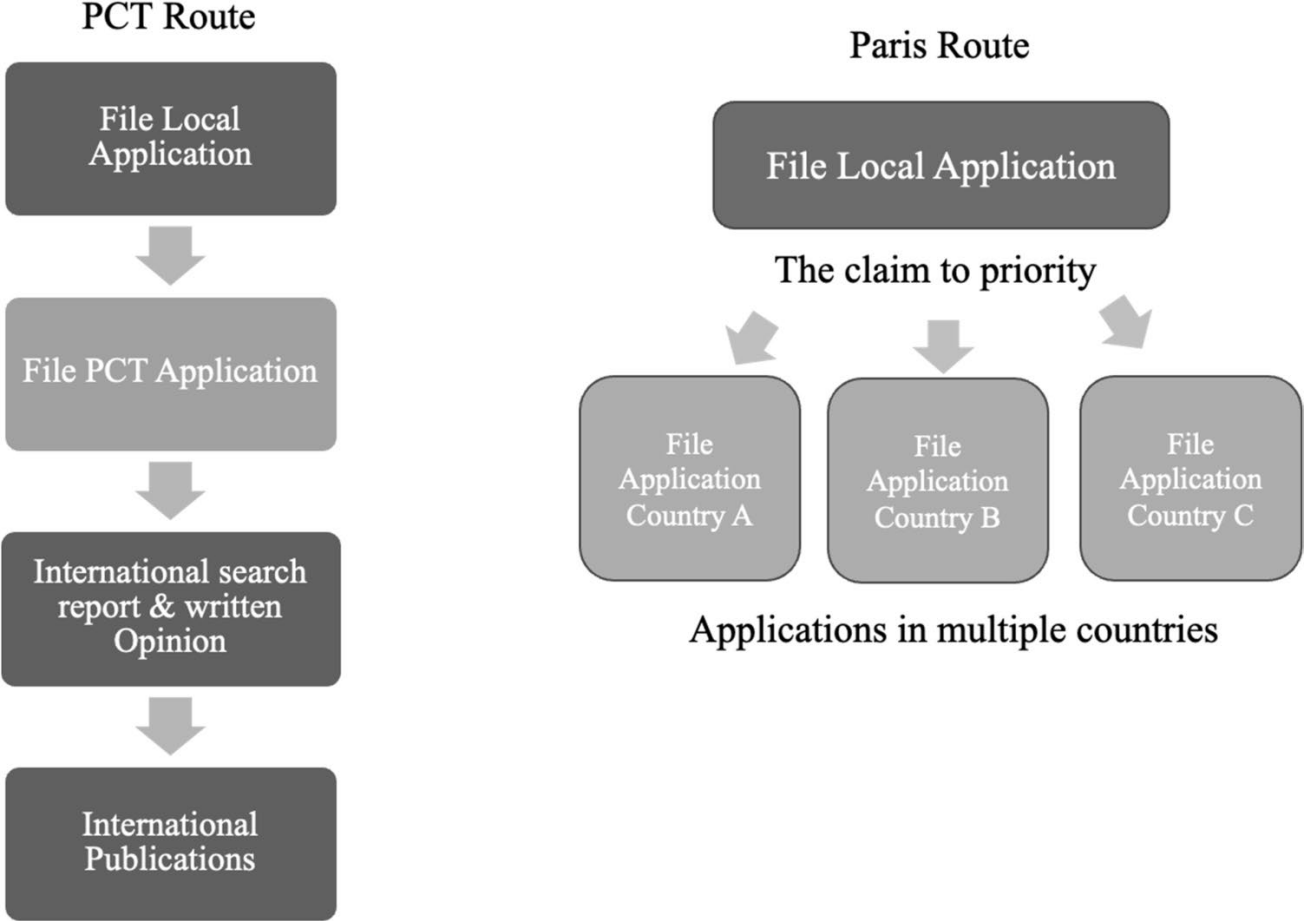
International application: comparison of Paris and PCT Route

The another way to obtain a foreign patent is, so-called “Paris route,” which involves direct filing of a foreign application using a national application. Unless the application is filed only in one country, the PCT application is more common and offers more advantages. Figure 5 summarizes the procedural differences between the PCT application and Paris Route application.

### How much does it cost to file a PCT application?

The total cost varies by case but is generally higher than a national application [5]. A PCT application incurs international filing fees. When transitioning to national phase, additional costs arise from translating and formatting the application for each country. If an office action (e.g., a notice of refusal) is issued, legal costs increase further. Filing in multiple countries can cost tens of thousands of Euros [5].

Given the high cost, universities and researchers typically seek company support when filing foreign patents. If a company sees potential for large-scale business in a country, they may fund the patent application.

### What is "entry to national phase"? Which countries should I choose?

“Entry to national phase” refers to selecting specific PCT member countries in which to pursue patent protection after the international phase.

Each selected country will conduct its own examination before granting a patent. Selection should be based on strategic business and manufacturing considerations, including:

- Countries where large-scale commercialization is planned.
- Countries that will serve as production hubs.
- Countries where manufacturing or part procurement will take place.
- Countries that will be distribution centers for related products.
- Countries where you wish to prevent counterfeit products from appearing.

Since international patenting is expensive, prioritizing key markets is crucial for cost-effectiveness and business viability.

## The IP mindset

### Rights are independent, and territorialism is the principle

Is the IP system the same worldwide? The answer is, of course, no. The IP protection system is based on the principle of national independence, meaning that intellectual property rights are territorial. However, differences in national laws could disadvantage rights holders. To mitigate this, international treaties and agreements harmonize IP systems to some extent. Since research activities are increasingly borderless, securing rights not only in the country of origin but also in other jurisdictions is crucial. Utilizing various international application systems is essential for effective protection.

### Medical practice and industrial applicability

Each country has a different approach to the patentability of medical procedures. In the United States, medical procedures can be patented, (“Method Patent”) but enforcement against doctors and medical institutions is restricted. In contrast, under the European Patent Convention (EPC, Article 52(4)) and Japanese patent law (Article 29, Paragraph 1), methods for operating, treating, or diagnosing humans are considered nonindustrial and thus not patentable. The concern is that allowing patents on medical procedures could discourage doctors from performing necessary treatments.

However, with rapid advancements in medical technology, the demand for patent protection in this field has grown. To balance innovation with public interest, patent protection is now granted to certain medical-related technologies, such as control methods for medical equipment, while still excluding direct medical procedures performed by doctors.

### The European Unitary Patent and the Unified Patent Court

The Unified Patent Court (UPC) opened in Europe in June 2023, marking the full launch of the European Unitary Patent System. Previously, the EPC established a unified patent grant procedure, simplifying and reducing the cost of obtaining patent protection across contracting countries. However, the resulting patents still required validation and enforcement in individual countries.

The new Unitary Patent System introduces a single patent with unitary effect across all UPC-participating countries. This system streamlines patent protection across Europe, making it more efficient and cost-effective. Researchers and companies engaged in R&D in Europe should take full advantage of this system.

### The IP mindset

Research activities demand substantial time, funding, and persistent effort. The innovations resulting from these endeavors can be truly unique and valuable. In the current era, it is important not only to focus on patents but also to consider other forms of IP, such as designs, trademarks, and copyrights—particularly concerning AI developments. The IP system should not be seen merely as a defensive tool; its true purpose is to accelerate innovation rather than hinder it. We hope that an ‘IP mindset’ will be cultivated among researchers worldwide.

## Summary

The EAES IP Protection Guide offers a comprehensive overview of basic IP knowledge tailored specifically for surgeons, engineers, and researchers involved in medical device innovation. Since collaboration between clinicians and industry is indispensable for surgical innovation, safeguarding IP is essential to ensure the translation of novel ideas into clinical products without being prematurely disclosed or exploited.

This guide was developed by leading experts within the EAES Technology Committee and addresses the current gaps in IP awareness revealed by an EAES survey [1], in which 60% of respondents reported sharing ideas without IP protection. It covers key IP concepts, including patents, design rights, trademarks, and NDAs, as well as outlines when and how to disclose inventions. We also highlight here the importance of early engagement with Tech Transfer Offices and the nuances of joint research agreements with industry partners.

Additionally, the guide clarifies the patent application process including costs, timelines, and ownership principles, especially for employee inventions, and emphasizes the importance of obtaining IP rights in multiple countries, international patent strategies via the PCT, and the newly introduced European Unitary Patent system.

Finally, the guide supports cultivating an “IP mindset” among surgical innovators, not only as a legal formality, but also as a proactive strategy to protect and promote translational research and patient-centered innovation across global markets.
